# Dose-Dependent Adverse Effects of Salinomycin on Male Reproductive Organs and Fertility in Mice

**DOI:** 10.1371/journal.pone.0069086

**Published:** 2013-07-01

**Authors:** Olajumoke Omolara Ojo, Smrati Bhadauria, Srikanta Kumar Rath

**Affiliations:** Toxicology Division, CSIR-Central Drug Research Institute, Lucknow, India; University of Colorado, United States of America

## Abstract

Salinomycin is used as an antibiotic in animal husbandry. Its implication in cancer therapy has recently been proposed. Present study evaluated the toxic effects of Salinomycin on male reproductive system of mice. Doses of 1, 3 or 5 mg/kg of Salinomycin were administered daily for 28 days. Half of the mice were sacrificed after 24 h of the last treatment and other half were sacrificed 28 days after withdrawal of treatment. Effects of SAL on body and reproductive organ weights were studied. Histoarchitecture of testis and epididymis was evaluated along with ultrastructural changes in Leydig cells. Serum and testicular testosterone and luteinizing hormones were estimated. Superoxide dismutase, reduced glutathione, lipid peroxidation, catalase and lactate dehydrogenase activities were measured. Spermatozoa count, morphology, motility and fertility were evaluated. Expression patterns of steroidogenic acute regulatory protein (StAR) and cytochrome P450 side chain cleavage proteins (CYP11A1) were assessed by Western blotting. Salinomycin treatment was lethal to few mice and retarded body growth in others with decreased weight of testes and seminal vesicles in a dose dependent manner. Seminiferous tubules in testes were disrupted and the epithelium of epididymis showed frequent occurrence of vacuolization and necrosis. Leydig cells showed hypertrophied cytoplasm with shrunken nuclei, condensed mitochondria, proliferated endoplasmic reticulum and increased number of lipid droplets. Salinomycin decreased motility and spermatozoa count with increased number of abnormal spermatozoa leading to infertility. The testosterone and luteinizing hormone levels were decreased in testis but increased in serum at higher doses. Depletion of superoxide dismutase and reduced glutathione with increased lipid peroxidation in both testis and epididymis indicated generation of oxidative stress. Suppressed expression of StAR and CYP11A1 proteins indicates inhibition of steroidogenesis. Spermatogenesis was however observed in testis 28 days after Salinomycin withdrawal. The results indicate reversible dose-dependent adverse effects of Salinomycin on male reproductive system of mice.

## Introduction

Salinomycin (SAL), an ionophore has been shown as an effective anti-cancer agent [1]. This monocarboxylic polyether is produced by *Streptomyces* *albus* [2]. SAL is commonly used as an antiprotozoal agent against coccidial parasites in poultry farming and as a growth promoter in chicken, pig and ruminants [3]. It has been demonstrated that SAL is more than 100 times efficient than paclitaxel in eliminating human cancer stem cells (CSCs) [1]. Cancer stem cells of blood, breast, brain, bone, skin, liver, bladder, ovary, prostate, colon and pancreas display numerous mechanisms of resistance to chemotherapeutics and radiation therapy leading to long-term tumour recurrence and metastasis [4]. Recent evidence shows that SAL is able to arrest cell cycle progression, induce apoptosis, break mitochondrial membrane potential, reverse multidrug resistance (MDR) and act synergistically with other anticancer drugs [5]. Its ability to kill cancer stem cells and apoptosis-resistant cancer cells made SAL unique and therefore, considered as a novel anticancer agent [6]. Pilot studies in humans and in mice carrying xenografts of tumors showing resistance through different mechanisms also showed promising antitumor effects of SAL [7]. SAL effectively eliminates CSCs [1] and induces partial regression of chemotherapy-resistant cancers [7]. The clinical potential of SAL has further increased as its amide and benzotriazole ester derivatives also showed antiproliferative activity against CSCs [8]. However, a number of incidences of toxicity had earlier been reported where SAL was accidentally fed or ingested in higher doses in different animals [9–20]. Though, SAL has been confirmed to have great potential for cancer treatment, its adverse effects need detailed investigation. As chemotherapy drugs are more likely to lead to infertility and have lasting effects on reproductive health it was thought opportune to evaluate SAL toxicity on male reproductive system of mice; an excellent model to monitor the potential damage induced by chemical agents [21].

## Materials and Methods

### Chemicals and reagents

Salinomycin (≥98%), Dimethyl sulphoxide (DMSO), Nitro blue tetrazolium (NBT), Nicotinamide adenine dinucleotide phosphate reduced tetrasodium salt (NADPH), Nicotinamide adenine dinucleotide (reduced) disodium salt, (NADH), Oxidized Glutathione, Glutathione (reduced), 2-Thiobarbituric acid, Potassium dichromate, Acetone, Ethanol, Benzene, Formaldehyde, Hematoxylin and Eosin, Toluidine blue, 5,5, Dithiobis (2-nitrobenzoicacid),1-Chloro-2,4-dinitrobenzene, Protease inhibitor cocktail, N,N,N’,N’-Tetra methyl ethylenediamine (TEMED), Acrylamide, N,N’-Methylene bisacrylamide, Ponceau S, Ammonium persulphate (APS), Bromophenol blue (BPB), Sodium dodecyl sulphate (SDS), Osmium tetroxide, Paraformaldehyde, Glutaraldehyde, Phenazine methosulfate were purchased from Sigma Chemical CO (St Louis MO,USA). Lactate dehydrogenase kit was purchased from Merck (Spain), testosterone ELISA kit was purchased from DRG (Germany). The antibodies of CYP11A1, StAR and β-actin were purchased from Santa Cruz Biotechnology (Santa Cruz, CA, USA).

### Animals, grouping and treatments

The protocol was approved by the Institutional Animal Ethics Committee of Central Drug Research Institute, Lucknow. This study was carried out in strict accordance with the recommendations in the Guide for the Care and Use of Laboratory Animals of the Institute. The IAEC clearance number is 109/10/Toxicol/IAEC.

Male and female balb/c mice weighing about 20 g were obtained from the Laboratory Animal Division of the Institute. The animals were maintained under standard conditions of humidity (50 ± 5%), temperature (25 ± 2^o^C) and dark and light cycles (12 h each) with free access to food and water. Male mice were divided into eight groups of ten animals each and treated intraperitoneally as follows;

Group I: Vehicle-treated control;
Group II: SAL, 1mg/kg/day for 28 days;
Group III: SAL, 3mg/kg/day for 28 days;
Group IV: SAL, 5mg/kg/day for 28 days;
Group V: Vehicle-treated control;
Group VI: SAL, 1mg/kg/day for 28 days;
Group VII: SAL, 3mg/kg/day for 28 days;

Group VIII: SAL, 5mg/kg/day for 28 days.

Groups I, II, III and IV were sacrificed 24 hour (h) after the last treatment. Groups V, VI, VII and VIII were sacrificed 28 days after drug withdrawal; fertility was checked before sacrifice. Females were used in fertility test.

### General observations, body and organ weights

Mice were observed daily for behavioural changes. Body weight was recorded daily prior to administration of SAL with the help of a mono pan balance. At autopsy, testes, epididymes and seminal vesicles were removed, blotted free of blood and adhering tissues and weighed.

### Serum Testosterone (T) and Luteinizing Hormone (LH) concentrations

The blood samples were drawn from the heart prior to autopsy and serum was separated by centrifugation and stored at -20^o^C until testosterone and luteinizing hormone assays were performed by enzyme-linked immunosorbent assay (ELISA) using the manufacturer’s instructions.

### Testicular Testosterone (T) and Luteinizing hormone (LH) concentrations

The testicular testosterone and luteinizing hormone levels in three mice from each group were measured [22]. Briefly, testicular proteins were extracted with phosphate buffer (50 mM, pH 7.4) and centrifuged at 10,000 g for 20 min. The supernatant was used to estimate T and LH levels using ELISA, and were expressed in ng/ml.

### Biochemical estimations in tissue samples

Testis and epididymis from each mouse (n=5) were stored at -80^o^C for different biochemical assays of lipid peroxidation: malonialdehyde (MDA), lactate dehydrogenase (LDH), glutathione (GSH), superoxide dismutase (SOD) and catalase (CAT). Protein quantity was estimated according to Lowry’s method [23]. 10% tissue homogenates (w/v) were prepared in chilled 100 mM Tris-HCL buffer (pH 7.4) using Cole Parmer tissue homogenizer. The values were expressed per mg of protein.

### Lipid peroxidation (LPO)

The lipid peroxidation was estimated by a spectrophotometric method in terms of thiobarbituric acid reactive substances. Briefly, one volume of homogenate was mixed with two volumes of stock solution (15% w/v trichloroacetic acid in 0.25 N HCL and 0.375% w/v thiobarbituric acid in 0.25 N HCL) in a centrifuge tube, vortexed and heated for 15 min at 95^o^C in water bath. The mixture was cooled and centrifuged at 5000 rpm for 5 min and the absorbance of the supernatant was read at 532 nm [24].

### Lactate dehydrogenase (LDH) activity

Lactate dehydrogenase was assayed according to manufacturer’s instructions. Briefly,10 µl of 10% homogenate of the tissues (w/v) and 300 µl of the working reagent (thermally mixed R_1_ and R_2_) were incubated at 37^o^C for 1 min. Reagent one (R_1_) was a mixture of 80 mmol phosphate buffer pH 7.8 and 0.6 mmol of pyruvate while reagent two (R_2_) was 0.18 mmol/L NADPH. The two reagents were mixed thoroughly and served as the working reagent. Absorbance at 340 nm was read at 1min interval for 3 min.

### Superoxide dismutase (SOD) activity

Superoxide dismutase (SOD) activity was estimated by a spectrophotometric method. Assay mixture containing sodium pyrophosphate buffer (pH 8.3, 0.052M), phenazine methosulfate (186 µM), nitroblue tetrazolium (300 µM) and NADH (780 µM) were diluted with appropriate enzyme in total volume of 3 ml. The mixture was incubated at 37^o^C for 90 sec and reaction was stopped by addition of glacial acetic acid. The reaction mixture was mixed vigorously by adding n-butanol and allowed to stand for 10 min before the collection of butanol layer. The intensity of chromogen in butanol was measured at 520 nm [25].

### Catalase (CAT) activity

Catalase activity was quantified by measuring the decomposition of hydrogen peroxide (H_2_O_2_). Assay mixture consisting of 0.01M phosphate buffer (pH 7), 0.2 M hydrogen peroxide and tissue homogenate was incubated at 37^o^C for 1 min. The reaction was stopped by addition of potassium dichromate (5% w/v) and acetic acid. The remaining hydrogen peroxide was determined by measuring chromium acetate after heating the assay mixtures in a boiling water bath for 15 min. The absorbance was read at 570 nm [26].

### Glutathione (GSH) content

Glutathione (GSH) content was estimated by centrifuging an aliquot of 10% homogenates of the tissues in 100 mM Tris-HCL buffer (pH 7.4) containing 0.16 M KCL at 1000 g for 5 min. The supernatant was used to measure the rate of reduction of 5’ 5’-dithiobis-(2 nitrobenzoate) to 2-nitro-5 thiobenzoate. The absorbance was read at 412 nm. Glutathione content was expressed in µM/mg protein [27].

### Histology of testis and epididymis

The testis and epididymis of five mice of each group were fixed in 10% formal saline, dehydrated in ethanol series and embedded in paraffin wax. Serial sections of 5 µm thickness were cut using a microtome and stained with haematoxylin-eosin. Sections were observed using a light microscope (Leica, Germany).

### Ultrastructure of testis

The ultrastructure of testis focusing on Leydig cells were studied [28]. Small pieces of testes were cut and fixed in 2.5% glutaraldehyde and 2% paraformaldehyde in 0.1M sodium phosphate buffer (pH 7.3) for 12 h at 4^o^C. After a buffer wash, the samples were fixed in 1% osmium tetroxide in 0.1M phosphate buffer for 1 h at 4^o^C. The samples were then dehydrated in an ascending grade of acetone, infiltrated and embedded in araldite CY 212 (TAAB, UK). Sections (1 µm) were cut with an ultramicrotome, mounted on clean glass slides, stained with aqueous toluidine blue and observed under a light microscope for gross observation of the area and quality of the tissue fixation. For electron microscopic examination, thin sections of grey-silver colour interference (70-80 nm) were cut and mounted onto 300 mesh-copper grids. Sections were then stained with alcoholic uranyl acetate and alkaline lead citrate, washed gently with distilled water and observed under a Morgagni 268D transmission electron microscope (FEI Company, Netherlands) at an operating voltage of 80 kV. Images were digitally acquired by using a CCD camera (Mega view III, FEI Company) attached to the microscope.

### Sperm morphology, count and motility

Cauda epididymidis was removed from each mouse and cleaned off from the epididymal fat pad, and minced in a pre-warmed Petri dish containing 500 µl phosphate buffer saline solutions (PBS, pH 7.4) at 37^o^C. Sperm motility was estimated by putting a drop of sperm suspension on a clean slide and covered with a cover slip and analysed by the computer assisted sperm analyser (CASA) by Hamilton, Thorne. The motility was expressed as percentage incidence [29]. For sperm count, an aliquot of this suspension was charged into the Neubauer’s counting chamber and the spermatozoa were counted under light microscope. Total sperm count was calculated as the average of the spermatozoa count (N) in each chamber X multiplication factor (10^6^) X dilution factor and was expressed in millions/ml [30]. The sperm morphology was also evaluated [21]. Briefly, a smear of sperm was made on a clean slide and stained with haematoxylin and eosin and were examined under a light microscope with an oil immersion lens. The morphology of spermatozoa was scored according to Qureshi et al. [31].

### Expression of StAR and CYP11A1 proteins by Western blotting

Total proteins were isolated from testes of mice using urea lysis buffer. 50 mg of total protein from each sample was separated on a 15% SDS-PAGE and transferred on to nitrocellulose membrane for immunodetection by a semi-dry electro-blotting apparatus (GE Health, UK). The membrane was blocked in 5% non-fat dry milk in phosphate buffered saline tween-20 (PBST) for 1 h at room temperature and incubated with primary antibodies of CYP11A1 and StAR at a dilution of 1:1000 in 2.5% non-fat dry milk in PBST overnight, followed by another incubation of horseradish peroxidase-conjugated goat anti rabbit secondary antibody at a dilution of 1:10,000. Immunoblots were reprobed with β-actin monoclonal antibody to confirm equal amount of protein loading. The expression levels of CYP11A1, StAR and β-actin, detected by immunoblotting, were quantitated using the program IMAGE (National Institutes of Health) for the integrated density of each band. Quantitative Western blot data were calculated from densitometric analysis of bands with the NIH imageJ software. The values were normalized to β-actin, which served as a loading control.

### Male fertility and dominant lethality

Male fertility was checked in mice of groups V, VI, VII, and VIII after 28 days of SAL treatment according to standard method [32,33]. Each male was caged with one female per week for four weeks. The females were at proestrous when allowed with the males. Each female was checked for vaginal plug daily for confirmation of mating. Following confirmation of mating, females were separated from males. The females were sacrificed on 13 days of mating to check implantation. The number of pregnant mice was recorded to determine percent of fertility [34]. The incidence of pregnancy was established after counting the number of implants. The dead implants per female were determined to obtain the post-implantation loss [35]. Fertility index was calculated by the ratio of the number of pregnant females to number of females cohabited with males multiplied by 100.

### Statistical analysis

All statistical comparisons between the groups were made using analysis of variance (ANOVA) by Prism statistics software. Results were presented as mean ± SEM (Standard Error Mean). Values of *p* < 0.05 were considered as statistically significant.

## Results

### General observations

**Figure 1:**
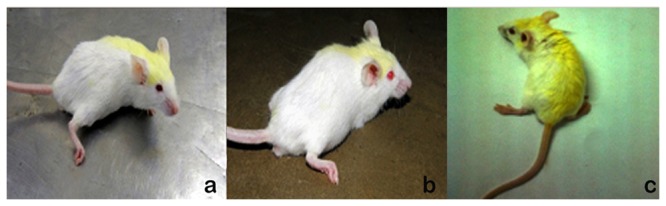
Mice treated with SAL a) 1mg/kg; b) 3mg/kg and c) 5mg/kg. Note the hind limb paralysis.

The earliest toxic signs observed in few SAL treated mice were weakness, salivation, diarrhoea and paralysis of hind limb (Figure 1). One mouse from group II and two mice each from groups III, IV, VII and VIII died between fifth and tenth day of intraperitoneal administration of SAL.

### Body and reproductive organ weights

SAL treatment for 28 days reduced the body weight gain significantly in mice of all the treatments (II, III, IV, VI, VII and VIII) in comparison to controls (Table 1). The weights of testis, epididymis and seminal vesicle also decreased after 3 and 5 mg of SAL treatment. The weight of these organs did not recover even 28 days after SAL withdrawal.

**Table 1:** Effect of SAL on body and reproductive organ weights.

Groups & Treatment (mg/kg/day)	Body weight (g)		Reproductive Organs weight (mg/100g of body weight)		
	Pre-dose	Post-dose	Testes	Seminal vesicles	Epididymes
Group I (Control)	20.02 ± 0.23	28.04 ± 1.95	1.06 ± 0.10	0.94 ± 0.09	0.13 ± 0.02
Group II (1mg/kg)	19.02 ± 0.32	22.90 ± 0.90***	1.01 ± 0.04	0.74 ± 0.15	0.11 ± 0.01
Group III (3mg/kg)	18.26 ± 0.30	20.58 ± 0.56***	0.98 ± 0.09**	0.67± 0.04***	0.09 ± 0.01*
Group IV (5mg/kg)	22.08 ± 1.26	22.92 ± 0.73***	0.60 ± 0.02***	0.59± 0.03***	0.09 ± 0.01*
Group V (Control)	20.00 ± 0.34	27.33 ± 1.74	1.01 ± 0.04	1.37±0.04	0.14 ± 0.01
Group VI (1mg/kg)	20.50 ± 0.23	24.18 ± 1.60*	0.75 ± 0.07*	1.23 ± 0.13	0.12 ± 0.01
GroupVII (3mg/kg)	23.91 ± 0.33	23.18 ± 1.05**	0.68 ± 0.03**	0.98 ± 0.04**	0.11 ± 0.01
GroupVIII (5mg/kg)	19.36 ± 0.19	19.85 ± 0.31***	0.55 ± 0.02***	0.84 ± 0.06**	0.10 ± 0.01*

Note: *, **, and *** indicate significant difference as compared to controls at (*p*<0.05), (*p*<0.01), and (*p*<0.001) respectively.

### Testosterone and luteinizing hormone concentrations

Serum testosterone level increased after 28 days of SAL treatment at a dose of 5 mg but then decreased 28 days after withdrawal of treatment. Testicular testosterone level was found to be reduced in all SAL treated mice. Significant decrease was also seen even after withdrawal of SAL treatment. The serum luteinizing hormone concentration showed increase in 3 and 5 mg SAL treated mice. The level was still elevated in 5mg SAL treated mice and even after 28 days of withdrawal. A dose-dependent depletion of testicular LH was seen in mice following 28 days SAL treatment and even after withdrawal of the treatment (Table 2).

**Table 2:** Effect of SAL on testosterone and Luteinizing hormone levels (ng/ml) in serum and testes of mice.

	Serum		Testis	
Groups &Treatment (mg/kg/day)	Testosterone (ng/ml)	Luteinizing Hormone (ng/ml)	Testosterone (ng/ml)	Luteinizing Hormone (ng/ml)
Group I (Control)	2.71 ± 0.05	1.99 ± 0.05	0.34 ± 0.00	6.93 ± 0.31
Group II (1mg/kg)	3.21 ± 0.01	2.93 ± 0.13	0.24 ± 0.01**	5.29 ± 0.23
Group III (3mg/kg)	3.67 ± 0.05	4.06 ± 0.20**	0.22 ± 0.00**	3.75 ± 0.11**
Group IV (5mg/kg)	4.41 ± 0.11**	8.77 ± 0.08***	0.21 ± 0.00**	2.54 ± 0.17**
Group V (Control)	3.03 ± 0.02	2.39 ± 0.16	0.36 ± 0.01	5.07 ± 0.32
Group VI (1mg/kg)	2.77 ± 0.23	2.94 ± 0.12	0.23 ± 0.01	3.57 ± 0.09
GroupVII (3mg/kg)	2.52 ± 0.23	3.75 ± 0.32	0.17 ± 0.01	3.18 ± 0.17
GroupVIII(5mg/kg)	2.49 ± 0.01**	5.83 ± 0.25**	0.14 ± 0.01**	2.48 ± 0.25**

Note: **, and *** indicate significant difference as compared to controls at (*p*<0.01), and (*p*<0.001) respectively.

### Histology of testis and epididymis

**Figure 2:**
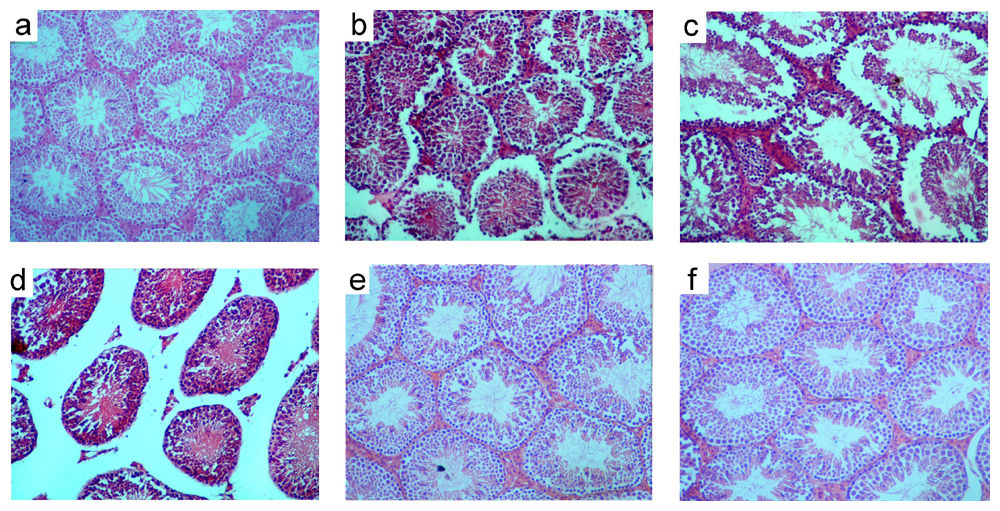
Transverse sections of testes of mice treated with SAL for 28 days (a) control (b) 1mg/kg (c) 3mg/kg and (d) 5mg/kg. Note the shrunken seminiferous tubules and vacuolation in the germinal epithelium (b, c & d) in SAL treated mice. Note the signs of recovery 28 days after withdrawal in treated mice; e (3mg/kg) and f (5mg/kg).

**Figure 3:**
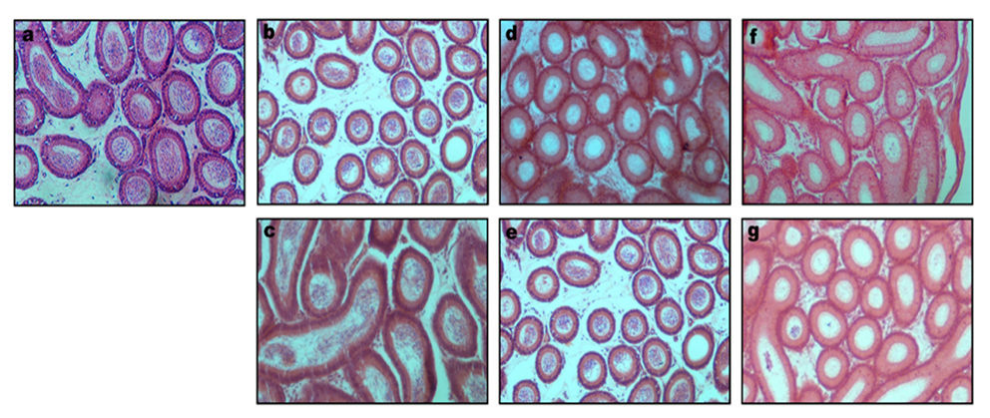
Transverse sections of caput epididymidis of mice following SAL treatment (a) control; (b) 1mg/kg; (c) 1mg/kg; (d) 3mg/kg; (e) 3mg/kg (f) 5mg/kg & (g) 5mg/kg. Note: Figures c, e & g are from the recovery groups.

**Figure 4:**
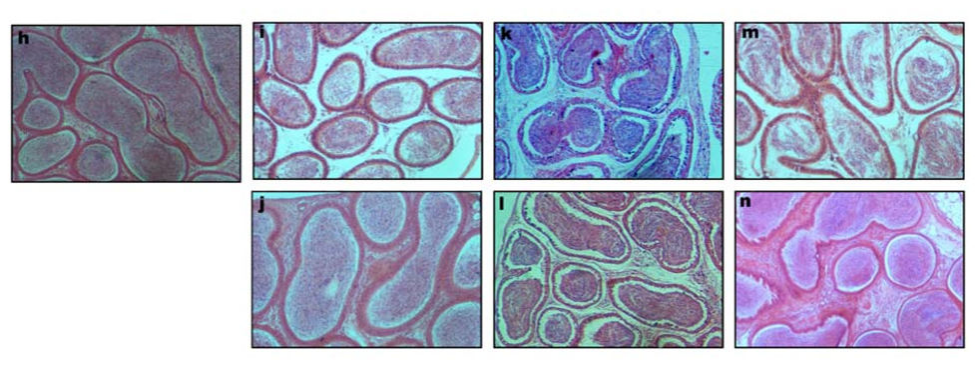
Transverse sections of cauda epididymidis of mice following SAL treatment (h) control; (i) 1mg/kg; (j) 1mg/kg; (k) 3mg/kg; (l) 3mg/kg; (m) 5mg/kg & (n) 5mg/kg. Note: Figures j, l & n are from the recovery groups.

SAL treatment induced various structural changes in the seminiferous tubules and interstitium of the testis. Disruption of spermatogenesis and shrinkage of the seminiferous tubules were observed throughout the cross sections of the testes in a dose-dependent pattern (Figure 2; b, c & d). Epithelial gaps, epithelial sloughing, and germ cell degeneration were observed in the seminiferous tubules frequently. In the seminiferous of mice groups VII and VIII, empty spaces in the epithelium were observed due to missing germ cells (Figure 2 c & d). SAL treatment at 5 mg led to severe vacuolization of the seminiferous tubules. However, normal seminiferous tubules with spermatogenesis and spermatozoa were observed in the testis of mice belonging to groups VII and VIII after SAL withdrawal (Figure 2; e & f). The disruption of epithelium with frequent occurrence of vacuolization and necrotic cells were observed in the cross section of epididymis with the disappearance of spermatozoa in the lumen of caput, corpus and cauda epididymides in SAL treated mice (Figures 3 & 4). However, epididymal epithelium appeared normal 28 days after SAL withdrawal.

### Ultrastructure of Leydig cells

**Figure 5:**
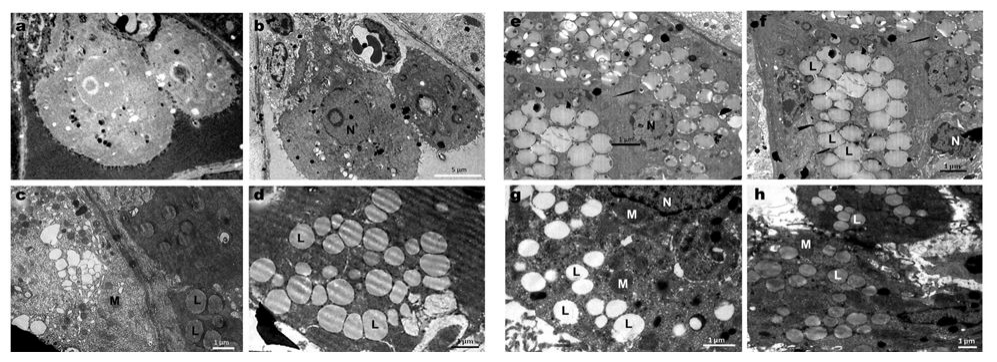
Electron micrographs of testes sections focusing on Leydig cells in controls (a & b) and SAL treated mice (c & d: I mg/kg; e & f: 3mg/kg and g & h: 5mg/kg). Note: Mitochondria-M; Nucleus -N; Lipid droplet -L.

The ultra structure of Leydig cells of SAL treated mice showed increased number of lipid droplets in comparison to controls (Figure 5 d, e, f, g & h). Cytoplasm of the cells was irregular, hypertrophied and partly disorganized with shrunken nuclei. The mitochondria were dark and condensed (Figure 5 g & h). Some degree of proliferation of smooth endoplasmic reticulum was also observed in all mice treated with 3 and 5 mg of SAL (Figure 5 e, f, g & h).

### Testicular and epididymal enzyme activities

SAL treatment resulted in significant increase (*p* <0.01) in the concentration of thiobarbituric acid reactive substances in both testis and epididymis tissue homogenates of mice. Similar effects were also seen in 5 mg dose group even after recovery (group VIII). SAL induced depletion in the protein level in testis and epididymis in a dose dependent manner. Oxidative stress was induced by SAL treatment as confirmed by the significant decrease (*p* <0.01) in GSH, CAT, LDH and SOD levels in both the testicular and epididymal tissues (Tables 3 & 4). However, significant increase (*p* <0.01) in reduced glutathione (GSH) was observed in the epididymis of mice following withdrawal of SAL treatment.

**Table 3:** Effect of SAL on activity of enzymes in the testis of mice.

Groups Treatment (mg/kg/day)	PRO (mg/ml)	CAT (µmol/min/mg protein)	SOD (U/mg protein)	GSH (µM/mg protein)	LPO (Umol/min/mg protein)	LDH (U/L)
Group I (Control)	7.49 ± 0.39	67.91 ± 2.61	7.70 ± 0.52	89.22 ± 9.40	0.87 ± 0.09	143.97 ± 23.16
Group II (1mg/kg)	5.59 ± 0.25**	61.73 ± 4.68	6.33 ± 0.50	80.97 ± 3.59*	1.63 ± 0.11**	124.87 ± 17.35**
Group III (3mg/kg)	4.63 ± 0.45***	49.66 ± 4.75*	5.19 ± 0.42**	51.00 ± 1.10***	2.47 ± 0.15***	96.54 ± 13.92***
Group IV(5mg/kg)	2.24 ± 0.17**	41.13 ± 3.99*	3.88 ± 0.45***	20.95 ± 3.91*	3.68 ± 0.32**	71.40 ± 8.21***
Group V (Control)	7.50 ± 0.53	70.84 ± 2.66	6.77 ± 1.20	77.70 ± 5.97	0.53 ± 0.06	151.26 ± 27.92
Group VI (1mg/kg)	5.64 ± 0.33*	62.94 ± 10.56**	4.83 ± 0.53	50.35 ± 3.27***	0.92 ± 0.12**	104.18 ± 25.44***
Group VII (3mg/kg)	3.47 ± 0.50**	52.91 ± 5.15***	4.50 ± 0.49**	39.28 ± 4.90***	2.27 ± 0.33***	81.63 ± 7.59***
Group VIII (5mg/kg)	2.33 ± 0.22**	34.68 ± 2.93***	3.47 ± 0.29**	18.56 ± 2.50***	2.97 ± 0.22***	68.46 ± 7.87***

Note: *, **, and *** indicate significant difference as compared to controls at (*p*<0.05), (*p*<0.01), and (*p*<0.001) respectively.

**Table 4:** Effect of SAL on activity of enzymes in the epididymis of mice.

Groups & Treatment (mg/kg/day)	PRO (mg/ml)	CAT (µmol/min/mg protein)	SOD (U/mg protein)	GSH (µM/mg protein)	LPO (Umol/min/mg protein)	LDH (U/L)
Group I (Control)	24.37 ± 1.38	69.39 ± 2.62	0.53 ± 0.31	23.12 ± 0.88	0.03 ± 0.00	0.89 ± 0.12
Group II (1mg/kg)	18.36 ± 1.10**	65.82 ± 0.92	0.50 ± 0.29	20.72 ± 2.32	0.17 ± 0.03**	0.52 ± 0.05***
Group III (3mg/kg)	13.82 ± 1.05***	40.25 ± 0.64***	0.41 ± 0.24**	10.99 ± 0.71***	0.34 ± 0.02***	0.43 ± 0.06***
Group IV(5mg/kg)	8.89 ± 0.71***	36.04 ± 1.22***	0.30 ± 0.17***	7.65 ± 1.70***	0.53 ± 0.08***	0.40 ± 0.09***
Group V (Control)	22.01 ± 0.57	63.56 ± 1.19	0.41 ± 0.21	16.40 ± 1.19	0.03 ± 0.00	0.81 ± 0.08
Group VI (1mg/kg)	10.63 ± 1.42***	38.88 ± 1.49***	0.30 ± 0.17*	28.90 ± 2.50***	0.41 ± 0.02***	0.63 ± 0.18***
Group VII (3mg/kg)	8.29 ± 1.67***	37.80 ± 3.68***	0.25 ± 0.15***	47.90 ± 1.45***	0.61 ± 0.04***	0.50 ± 0.11***
Group VIII (5mg/kg)	5.17 ± 0.58***	26.21 ± 7.68***	0.18 ± 0.10***	61.43 ± 13.73***	0.96 ± 0.10***	0.39 ± 0.02***

Note: *, **, and *** indicate significant difference as compared to controls at (*p*<0.05), (*p*<0.01), and (*p*<0.001) respectively.

### Sperm motility, count and morphology

**Figure 6:**
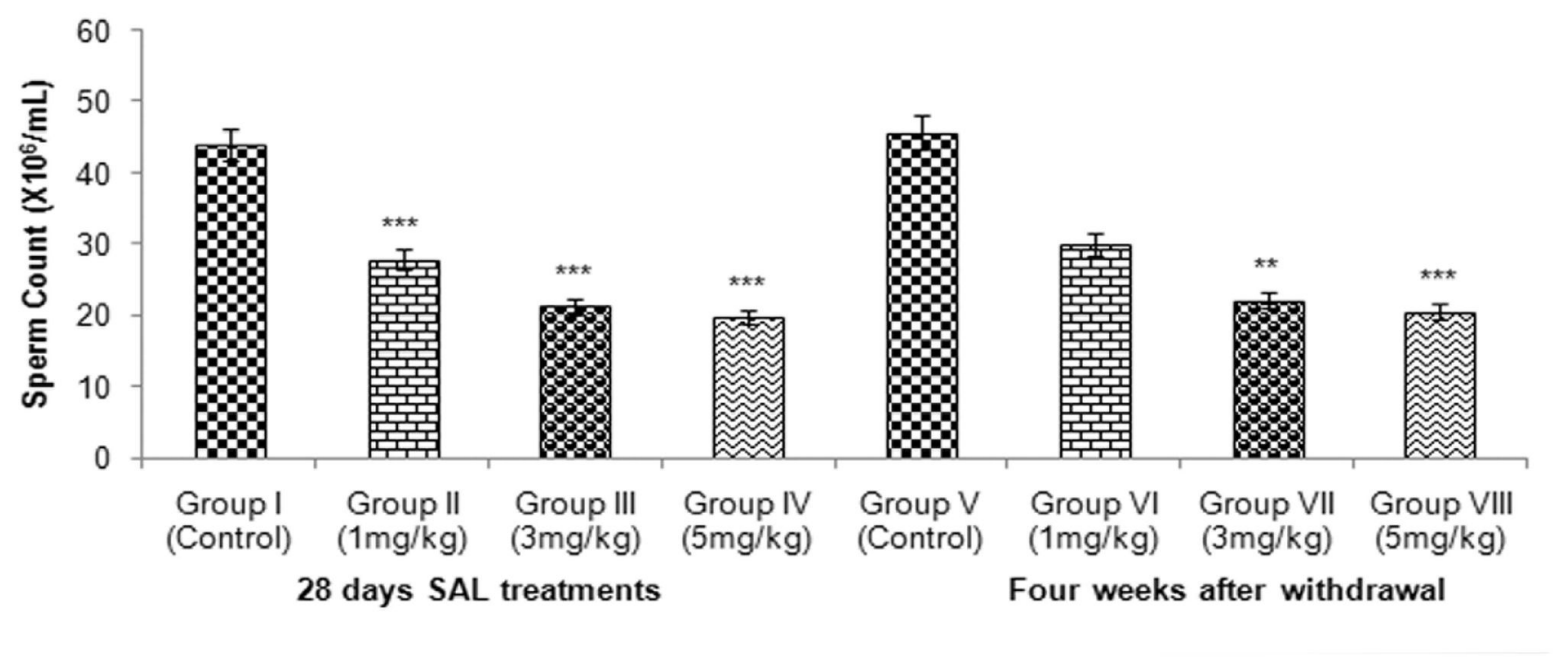
Effect of SAL on sperm count.

**Figure 7:**
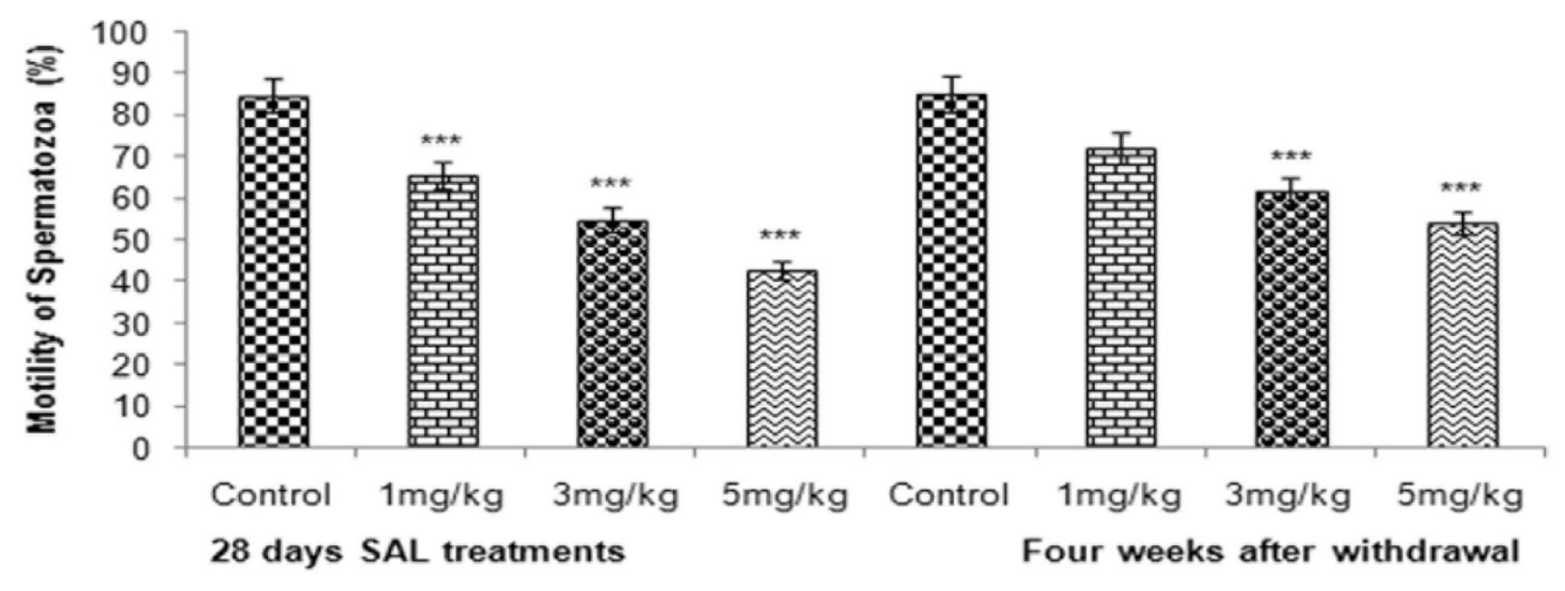
Effect of SAL on sperm motility.

**Figure 8:**
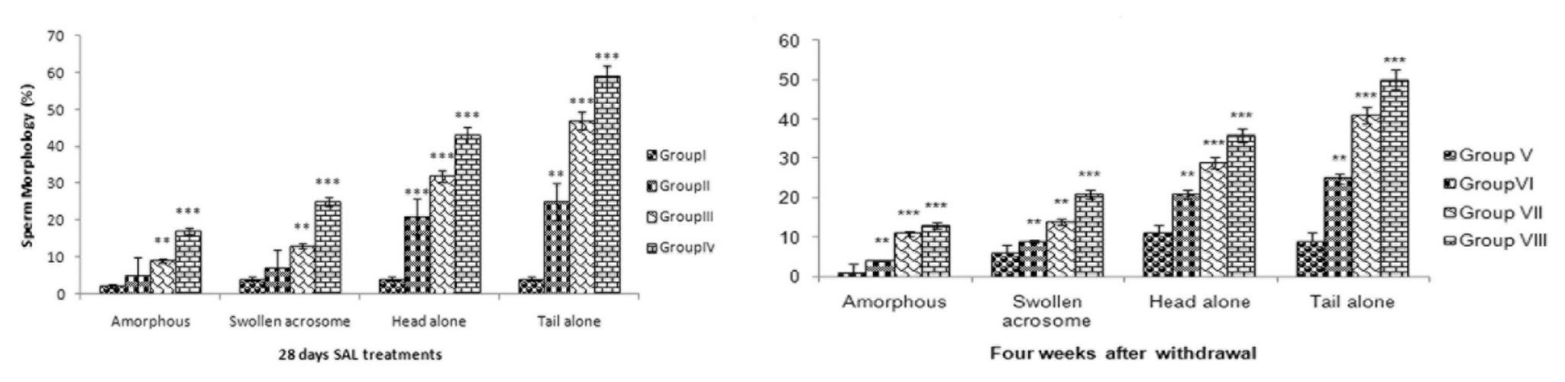
Effect of SAL on sperm morphology.

SAL treatment caused significant decrease (*p* <0.001) in epididymal sperm count and motility (Figures 6 & 7) across the SAL treatment groups. A marked increase (*p* <0.01) in abnormal morphology was observed in epididymal spermatozoa (Figure 8) in all SAL treated mice.

### Expression of StAR and CYP11A1 proteins

**Figure 9:**
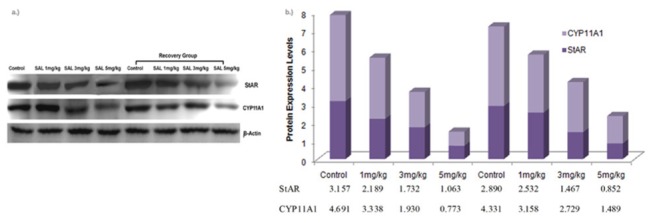
Expression levels of steroidogenic acute regulatory protein (StAR) and cytochrome P450 side-chain cleavage (CYP11A1) in the testis of mice (a) Western blot (b) densitometric quantitation of the Western blot.

Expression levels of CYP11A1 and StAR proteins in the testis of mice decreased significantly following SAL treatment for 28 days. Significant decrease was also seen in both proteins even after 28 days of withdrawal of SAL treatment (Figure 9).

### Male fertility and dominant lethality

The SAL treatment decreased fertility in male mice, as decreased rate of pregnancy and post implantation loss were seen in females mated to these males (Table 5). Infertility was observed in males treated with 3 and 5mg doses of SAL.

**Table 5:** Incidences of pregnancy and mean number of implants per female mated with SAL treated males.

Groups &Treatment (mg/kg/day)	Pregnant females	Male fertility index (%)	Average No of Implants/ female (mean ± S.E.M)		
			Total	Live	Dead
Group V (Control)	16/20	80	8.69 ± 0.29	8.38 ± 0.16	0.30 ± 0.13
Group VI (1mg/kg)	6/20	30	6.88 ± 3.08**	3.38 ± 1.74***	3.50 ± 1.34**
Group VII (3mg/kg)	0/20	0	0	0	0
Group VIII(5mg/kg)	0/20	0	0	0	0

Note: **, and *** Indicate significant difference as compared to control at (*p*<0.01), and (*p*<0.001) respectively.

## Discussion

There has been a search for new anticancer agents to treat cancer resistance throughout the globe. SAL, a broad spectrum antibiotic and a coccidiostat [36] has been found to counter tumour resistance and kills cancer stem cells [1] with better efficacy than the existing chemotherapeutic agents; paclitaxel and doxorubicin [1,37]. This refocused its importance for treatment of human cancers. However, a narrow margin of safety of SAL is a major cause of concern [38] and requires detailed toxicity evaluation. Present investigations were focused on SAL effects on male reproduction and fertility in mice as majority of cancer drugs demonstrated antifertility effects. Administration of SAL led to hind feet paralysis in few mice. Similar observations like tongue and pharyngeal paralysis in cattle [39] and a severe sensorimotor polyneuropathy with acute hind-limb paralysis in cat [40] had earlier been reported. Although there are many known causes for paralysis, drugs that interfere with nerve function cause paralysis very often. SAL might be one of them and needs further investigation. Weakness, diarrhoea and death of nine mice occurred after five to ten days of SAL administration across the groups and the effects were similar to those of chicken [41]. Reduced growth rate in mice following SAL treatment are very similar to the earlier reports in turkeys [42] and broilers [43–47]. These observations are critical and need further investigations.

A decrease in weight of testis and seminal vesicle observed in 3 or 5 mg/kg dose of SAL, indicated damage of reproductive organs with structural changes. Decrease in testis weight had been correlated with tubular atrophy along with germ cell death, and epithelial sloughing [48,49]. Antibiotics such as gentamycin and aflatoxin have also been reported to reduce sex organ weights in mice [50,51]. It is interesting to note that germ cells comprising of spermatogonia, spermatocytes, spermatids and spermatozoa were reduced in the seminiferous tubules of SAL treated mice which contributed to the weight of the testis. The adverse effects of SAL on the testis are comparable with that of polyphenols like gossypol. Similar dysfunctions in the testis including decreased weight, regressed seminiferous tubules, reduced number of spermatozoa with reduced motility were reported in many mammalian species following gossypol administration [52–54]. The vacuolated epithelium of epididymis and aggregation of necrotic cells in its lumen indicated abnormal spermatogenesis as well as lack of testosterone supply [55]. The decrease in weight of seminal vesicle and decrease in testicular testosterone further established SAL interference with androgen supply.

Increase in number of lipid droplets in the Leydig cell cytoplasm in SAL administered mice in a dose-dependent manner is indicative of cholesterol accumulation for testosterone synthesis. Hypertrophied Leydig cells with shrunken nuclei, disorganized cytoplasm, condensed mitochondria and proliferated smooth endoplasmic reticulum indicated impairment of functions like steroidogenesis resulting in reduced utilization of cholesterol. The use of free cholesterol in the process of steroidogenesis draws off substrate, thereby reducing the number of lipid droplets, whereas the blocking of this process would build up lipid droplets as cholesteryl ester storage. A declined secretory activity correlated with an increased number of lipid droplets, reduced sER and giant whorl-like smooth endoplasmic reticulum has been demonstrated in mouse Leydig cells following streptozotocin treatment [56,57]. Melatonin has also been shown to alter the ultrastructure of mouse Leydig cells and decrease nuclear volume [58].

To understand the mechanism of SAL interference in the suppression of testosterone, the expression of two important steroidogenic proteins controlling the rate limiting steps of steroidogenesis was studied in testis. Steroidogenic acute regulatory protein (StAR) transports cholesterol into inner mitochondria and cytochrome P450 side-chain cleavage (CYP11A1) catalyses the conversion of mitochondria cholesterol into pregnenolone to initiate the synthesis of steroids [59,60]. Down regulation of expression of these two mitochondria proteins and condensed mitochondria in the Leydig cells are indicative of impaired steroidogenesis following SAL exposure. SAL functions in different biological membranes, including cytoplasmic and mitochondrial membranes, as an ionophore with strict selectivity for alkali ions and a strong preference for potassium and it can disrupt Na^+/^Ca^2+^ exchange [61,62] leading to an increase in intracellular calcium levels [63,64]. Also, previous studies have demonstrated that Ca^2+^ influx and out flux should be tightly regulated to maintain the intracellular Ca^2+^ homeostasis; an alteration in the Ca^2+^ transport across the cell membrane could result in a drastic impact on steroidogenesis and spermatogenesis [65,66].

SAL treatment for 28 days significantly reduced the testosterone in the mouse testis. This might have resulted in infertility in males since testosterone essentially regulates sexual behaviour, accessory sex organ functions, epididymal sperm maturation, and spermatogenesis [67] and toxic agents which inhibit testosterone biosynthesis or secretion have profound effects on any of these processes required for the timely deposition of viable spermatozoa into the female reproductive tract. A similar finding was reported in rat after treatment with puromycin and cycloheximide [68]. Leydig cells produce the bulk of testicular testosterone and its biosynthetic enzymes are sequestered in Leydig cell mitochondria and smooth endoplasmic reticulum (sER) [69]. Adrenals also produce some amount of androgens in males [70]. Their synthesis in the gonads is controlled by the anterior pituitary hormones like LH [71]. High level of LH in serum indicates a reduction of testosterone in the testes resulting in absence of normal feedback [72] leading to a pituitary surge of LH. As LH level is high in serum and testis is unable to respond due to adverse effects of SAL, adrenal might have produced extra testosterone that resulted in raised level of testosterone in the serum.

A dose-dependent increase in lipid peroxidation (LPO) and depletion of enzymes such as superoxide dismutase, reduced glutathione, and lactate dehydrogenase following SAL treatment in both testis and epididymis clearly demonstrated failure of their antioxidant defence system. Decreased catalase level indicates accumulation of hydrogen peroxide. It may be noted that previous investigators reported that the reduction of GSH levels leads to elevation of LPO [73,74]. The present study also demonstrated that GSH level in testis and epididymis declined significantly in SAL treated mice. Similarly, Keshavarz and McDougald reported a significant elevation of LPO and depletion of serum GSH in broiler chicken following SAL poisoning [75]. Therefore, elevation of lipid peroxidation and depletion of GSH might be inducing oxidative stress by increasing the free radical generation in testis and epididymis resulting in disregulation of spermatogenesis and fertility [76].

Decrease in sperm count observed in the cauda epididymidis of SAL treated mice indicated cytotoxicity of SAL in the testis. The early germ cells in the spermatogenic cycle were affected by SAL and became abnormal thereby increasing the number of abnormal spermatozoa. Significant decrease in sperm motility indicated adverse effects of SAL on spermatozoa function probably through the structure and function of both testis and epididymis. The deficiency of glutathione might be one of the major causes [77]. Although the damaged germinal epithelium in the testis appears to be main reason for impaired sperm quality, oxidative stress in the epididymis might be the other reason. Increased lipid peroxidation in the testis might have also contributed to abnormality of spermatozoa resulting in infertility [78].

SAL triggered reduction in incidences of pregnancy and increased post-implantation loss. Incidences of decreased egg production, fertility and hatchability in broilers due to the administration of SAL, nicarbazin and narasin had earlier been reported [79–81]. The alterations in spermatogenesis resulting in abnormal spermatozoa having reduced motility could be correlated with the reduction in the number of implantations in the females mated with SAL treated males. The cause of reduced fertility in male mice following SAL administration could therefore be through interference with androgen supply. Signs of spermatogenesis in the seminiferous tubules in the testis of mice 28 days after SAL withdrawal indicated that adverse effects of SAL are reversible. It may be noted that the epididymal epithelium also showed signs of recovery following withdrawal of the treatment. However, SAL must be studied extensively before its use in cancer treatment.

## Conclusion

Intra peritoneal SAL administration at 1, 3 or 5 mg/kg for 28 days affects histoarchitecture of testis and epididymis, induces testicular dysfunction, fails spermatogenesis, suppresses steroidogenesis and leads to infertility in mice. Generation of oxidative stress and suppression of StAR and CYP11A1 protein expression may be responsible for the antifertility effects.
