# Correction: Dose-dependent adverse effects of salinomycin on male reproductive organs and fertility in mice

**DOI:** 10.1371/journal.pone.0226872

**Published:** 2019-12-19

**Authors:** Olajumoke Omolara Ojo, Smrati Bhadauria, Srikanta Kumar Rath

The image for Fig 3B is incorrectly duplicated as Fig 3E. The correct Fig 3 appears here.

**Fig 3:**
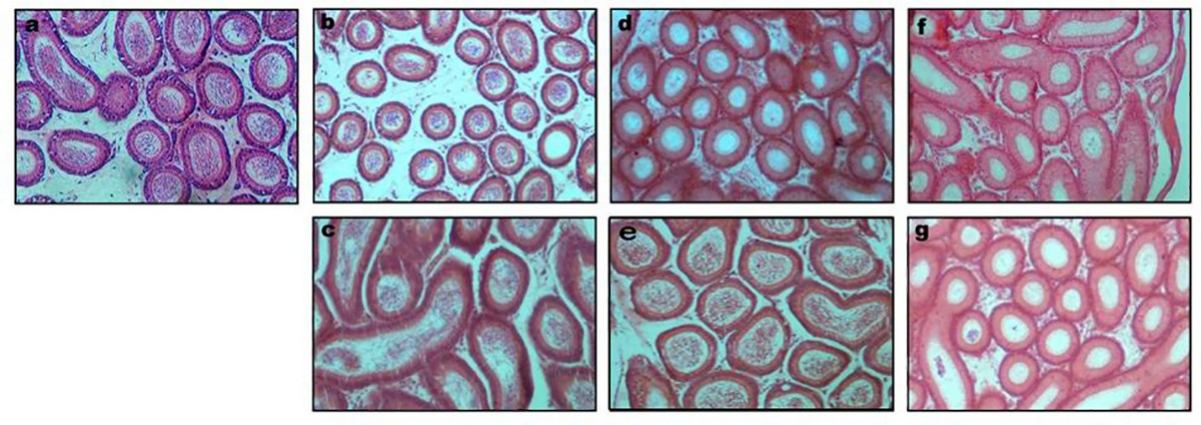
**Transverse sections of caput epididymidis of mice following SAL treatment (a) control; (b) 1mg/kg; (c) 1mg/kg; (d) 3mg/kg; (e) 3mg/kg (f) 5mg/kg & (g) 5mg/kg.** Note: Figures c, e & g are from the recovery groups.
